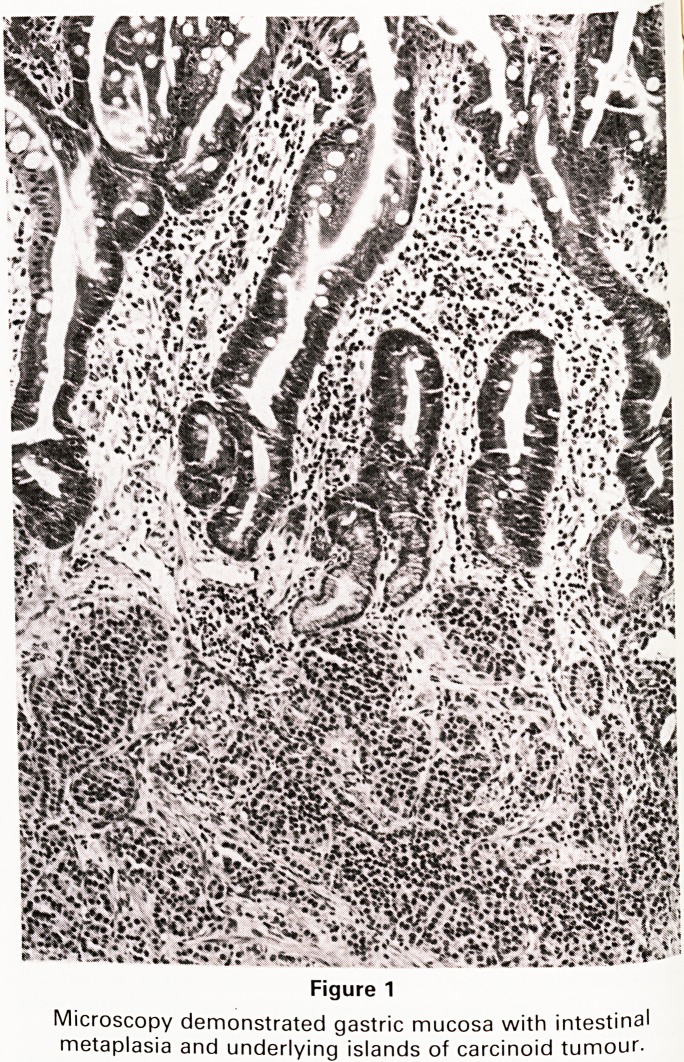# Gastric Carcinoid Presenting with Haematemesis

**Published:** 1987-05

**Authors:** C. F. M. Weston, D. A. Macdonald, A. C. Rikabi

**Affiliations:** Departments of Medicine, Surgery and Pathology, Bristol Royal Infirmary, Bristol, BS2 8HW; Departments of Medicine, Surgery and Pathology, Bristol Royal Infirmary, Bristol, BS2 8HW; Departments of Medicine, Surgery and Pathology, Bristol Royal Infirmary, Bristol, BS2 8HW


### Gastric carcinoid presenting with haemetemesis

**C. F. M. Weston, MRCP, D. A. Macdonald, MB, BS, A. C. Rikabi, MD.**
Departments of Medicine, Surgery and Pathology, Bristol Royal Infirmary, Bristol, BS2 8HW

*Case History*

A 57 year old man was admitted following episodes of epigastric pain, dizziness, melaena and rectal bleeding over the preceding 3 days. On direct questioning he admitted to having a chronic cough and shortness of breath on exertion, but no wheezing. His bowels were usually opened 3 times per day, passing formed stool. he had not experienced flushing. He smoked between 50 and 60 cigarettes per day and drank approximately 8 pints of beer per night. He had received no medication.

On admission he was pale with a pulse rate of 110 beats per minute and blood pressure of 110/70, there were no added heart sounds or murmurs and the venous pressure was not elevated. He was tender in the epigastrium and rectal examination revealed dark red blood.

The haemoglobin was 9.9 g/dl and plasma urea 23·3 mmol/L. He was resuscitated and a gastroscopy was performed. This revealed fresh blood in the stomach and an elevated round lesion with central ulceration in the distal body from which biopsies were obtained. The clinical impression was of leiomyoma. Histology demonstrated chronic inflammation of the lamina propria but no tumour. Meanwhile the patient suffered 2 episodes of significant haemetemesis (>500 ml of blood) and after further resuscitation underwent laparotomy and excision of the tumour, with a 3 cm margin of clearance. The operative findings were of an isolated tumour, 2 cm in diameter, with central ulceration where a blood vessel was visible. Histologically, the appearances were those of a gastric carcinoid which had not infiltrated the full thickness of the stomach wall and was completely excised (see illustration).

The patient made an uneventful recovery. Urinary 5H1AA and liver ultrasound scan were both normal.

**Figure 1**

Microscopy demonstrated gastric mucosa with intestinal metaplasia and underlying islands of carcinoid tumour.

### DISCUSSION

Carcinoid tumours in the stomach were first reported in 1923 in the German literature, and sporadically thereafter. Gastric carcinoid accounts for only 0.3% of all stomach neoplasms and a little over 2.0% of all carcinoids (1,2). Even excluding appendicular carcinoids, gastric carcinoids only account for 10.0% of the remainder (3). Pre-operative diagnosis is difficult because of variable radiographic appearance and the rarity of associated Carcinoid Syndrome or increased urinary 5H1AA level, though 5-hydroxytryptophan or serotonin excretion may be elevated.

Gastrointestinal haemorrhage as a presenting feature has been thought to be rare (4), but review of the literature reveals that, for gastric carcinoids at least, presentation with anaemia, melaena or haemetemesis is common. Thus bleeding was a feature in 4 of 8 cases reported by Balthazar (5), in 5 of 13 patients reviewed by Hines and Savage (6) and 4 of 5 cases reported by Lattes and Grossi (7). More specifically, haemetemesis was first reported in gastric carcinoid in 1936 (8) and in at least 4 other cases subsequently (9–12). In a retrospective review of 30 Swedish patients with gastric carcinoid, haemetemesis occured in 9, requiring emergency laparotomy in 4 cases (13).

This is not unexpected as carcinoid tumours of the stomach have been demonstrated to be ulcerated radiologically, histologically and macroscopically (5,13,14) and to be surrounded by an intense tumour "blush" angiographically.

### REFERENCES

1. GODWIN J. D. (1975) Carcinoid tumours. An analysis of 2837 cases. Cancer. **36**, 560–569.
2. MARTENSSON H., NOBIN A., SUNDLER F. (1983) Carcinoid tumours in the gastrointestinal tract: an analysis of 156 cases. Acta Chir Scand. **149**, 607–616.
3. CLEMENTS J. L., HIXSON Jr G. L., BERK R. N. et al. (1984) Gastrointestinal carcinoid tumours: an analysis of 104 cases. Mt Sinai J Med. **51**, 351–359.
4. WATKINS R. M. (1982) Gastrointestinal haemorrhage—an unusual presentation of carcinoid tumours. Postgrad Med J. **58**, 580–581.
5. BALTHAZAR E. J., MEGIBOW A., BRYK D., COHEN T. (1982) Gastric carcinoid tumours: radiographic features in 8 cases. AJR. **139**, 1123–1127.

*(continued on page 50)*

### Gastric carcinoid presenting with haemetemesis (Continued from page 40)

6. HINES C. R., SAVAGE J. L. (1955) Carcinoid tumours of the stomach. Ann Inter Med **43**, 859–867.
7. LATTES R., GROSSI C. (1956) Carcinoid tumours of the stomach. Cancer. **9**, 698–711.
8. ENTWISLE R. M. (1937) Carcinoid tumours of the stomach. Penn Med J. **40**, 1026–1028.
9. JONSSON S. O. (1949) Carcinoids of the small intestine and stomach. Acta Chir Scand. **98**, 390–395.
10. SCHERMAN V. E., HARA M., TRAFTON H. F. (1949) Massive haemorrhage from carcinoid of the stomach. J Miss Med Ass **46**, 175–177.
11. MARSHAK R. H., FRIEDMAN A. I. (1951) Carcinoids (argentaffinomas) of the stomach. AJR. **66**, 200–203.
12. HONIG L. J., WEINGARTEN G. (1974) A gastric carcinoid tumour with massive bleeding. Am J Gastroenterol. **61**, 40–41.
13. JOHANSSON H., WILANDER E. (1982) A clinical study of 30 gastric carcinoids. Uppsala J Med Sci. **87**, 135–142.
14. SIEGELMAN S. S., GOLD J. A., SIMON M., SOIFER I. (1969) Ulceration of intramural gastric neoplasms. Am J Dig Dis. **14**, 127–134.

## Figures and Tables

**Figure 1 f1:**